# *p16*^*INK4a *^hypermethylation and *p53*, *p16 *and *MDM2 *protein expression in Esophageal Squamous Cell Carcinoma

**DOI:** 10.1186/1471-2407-10-138

**Published:** 2010-04-13

**Authors:** Noushin Taghavi, Firouzeh Biramijamal, Masoud Sotoudeh, Hooman Khademi, Reza Malekzadeh, Omeed Moaven, Bahram Memar, Azadeh A'rabi, Mohammad Reza Abbaszadegan

**Affiliations:** 1 National Institute of Genetic Engineering and Biotechnology (NIGEB), Tehran, Iran; 2 Digestive Disease Research Center (DDRC), Tehran University of Medical Sciences, Tehran, Iran; 3 Division of Human Genetics, Immunology Research Center, Avicenna Research Institute, Mashhad University of Medical Sciences (MUMS), Mashhad, Iran; 4 Department of Pathology, Omid Hospital, MUMS, Mashhad, Iran

## Abstract

**Background:**

Tumor suppressor genes *p53 *and *p16*^INK4a ^and the proto-oncogene *MDM2 *are considered to be essential G1 cell cycle regulatory genes whose loss of function is associated with ESCC carcinogenesis. We assessed the aberrant methylation of the *p16 *gene and its impact on *p16*^*INK4a *^protein expression and correlations with *p53 *and *MDM2 *protein expressions in patients with ESCC in the Golestan province of northeastern Iran in which ESCC has the highest incidence of cancer, well above the world average.

**Methods:**

Cancerous tissues and the adjacent normal tissue obtained from 50 ESCC patients were assessed with Methylation-Specific-PCR to examine the methylation status of *p16*. The expression of *p16*, *p53 *and *MDM2 *proteins was detected by immunohistochemical staining.

**Results:**

Abnormal expression of *p16 *and *p53*, but not *MDM2*, was significantly higher in the tumoral tissue. *p53 *was concomitantly accumulated in ESCC tumor along with *MDM2 *overexpression and *p16 *negative expression. Aberrant methylation of the *p16*^*INK4a *^gene was detected in 31/50 (62%) of esophageal tumor samples, while two of the adjacent normal mucosa were methylated (P < 0.001). *p16*^*INK4a *^aberrant methylation was significantly associated with decreased *p16 *protein expression (P = 0.033), as well as the overexpression of *p53 *(P = 0.020).

**Conclusions:**

*p16 *hypermethylation is the principal mechanism of *p16 *protein underexpression and plays an important role in ESCC development. It is associated with p53 protein overexpression and may influence the accumulation of abnormally expressed proteins in *p53-MDM2 *and *p16-Rb *pathways, suggesting a possible cross-talk of the involved pathways in ESCC development.

## Background

Esophageal cancer is the fifth leading cause of cancer-related deaths in Iran [1]. It is also considered the second most common type of cancer in both males and females in Golestan province of northeastern Iran (Age Standardized Rate: 22.57 in males and 19.89 in females in 10^5 ^persons/year) (unpublished data). This region is located in the "Esophageal Cancer Belt," stretching from China westward through central Asia to northern Iran, where there is a high incidence of Esophageal Squamous Cell Carcinoma (ESCC)[2,3]. Although several environmental risk factors are proposed for ESCC, the specific underlying genetic alterations have not been well defined for this region to date [3,4].

Several genetic and epigenetic alterations are involved in esophageal carcinogenesis [5-7]. Investigation of alterations in oncogenes and tumor suppressor genes implicated in esophageal tumorigenesis may provide molecular markers for early diagnosis and therapeutic intervention[8].

Tumor suppressor genes *P53 *and *p16*^*INK4a *^and the proto-oncogene MDM2 (Murine Double Minute 2), are considered to be essential G1 cell cycle regulatory genes and whose loss of function is associated with cancer development [9].

In response to DNA damage, *p53 *protein induces G1 cell cycle arrest or apoptosis [10]. Abnormalities of this gene, such as gene mutation, can lead to the loss of regulation of cell growth, DNA repair, or apoptosis, all of which are responsible for carcinogenesis [11]. The product of the MDM2 gene, which is induced by p53, appears to play a critical role in regulating the level of the wild-type p53 protein. It can bind to and inactivate the transcriptional activity of p53, resulting in the abrogation of p53 anti-proliferative and apoptotic effects, and consequently the deregulation of cell overgrowth, which leads to tumor development [11-13]. Acting as a cyclin-dependent kinase inhibitor (CDKI), p16^*INK4a *^binds to and inhibits the activity of CDK4 and CDK6 and arrests the cell cycle in the G1/S phase in a p53-dependent pathway [14,15].

DNA methylation in the normally unmethylated promoter region of tumor suppressor genes, such as *p16*^*INK4a*^, is a critical mechanism for their inactivation and is commonly associated with the repression of gene transcription which promotes the development of cancers, including ESCC carcinogenesis [16-18]. Previous reports from Iran showed that *p16 *hypermethylation in the promoter region is a common mechanism for the inactivation of this gene in ESCC and gastric cancer development in Khorasan province of northeastern Iran [19,20]. Aberrant *p16 *hypermethylation is also suggested as a possible epigenetic risk factor in familial ESCC [21].

In addition, p53 alterations, including the mutation of p53, have been identified as a frequent event in ESCC development in northeastern Iran [22,23].

In the present study, we examined the methylation status of the *p16 *gene, in 50 ESCC patients using Methylation Specific PCR (MSP) assay. *p16*, *p53 *and *MDM2 *protein expression was assessed to identify the association of *p16*^*INK4a *^gene methylation with the expression of these cell cycle proteins in a population with a high incidence of ESCC in northeastern Iran.

## Methods

### Study population and sample collection

A total of 50 ESCC patients (ages ranging from 35-83 years) were recruited from May 2006 to June 2007, from among patients referred to the two main referral oncology centers in northeastern Iran: Atrak clinic, a referral center for upper GI cancers in Golestan province, and Omid Oncology Hospital, referral oncology hospital in northeastern Iran. It is estimated that approximately 95% of upper GI cancer patients in this region are referred to Atrak clinic [24]. Patients did not receive any adjuvant therapy (radiotherapy or chemotherapy) or blood transfusions. Follow up carried out 6 to 24 months afterwards. Demographic characteristics and information about social habits, including the smoking or consumption of cigarettes, the hookah and nass (a mixture of tobacco, ash, and lime) [4], were obtained by trained interviewers using a standard questionnaire. All patients underwent upper GI endoscopy. Tissue samples of esophageal tumor and macroscopically adjacent normal tissue from a site, remote from the tumor, were dissected and fixed in 70% alcohol and embedded in paraffin. The presence of normal and tumor tissue was assessed by histological evaluation. The Research Ethics Committees of Tehran University of Medical Sciences and Mashhad University of Medical Sciences approved the study design. All eligible patients signed a written informed consent prior to participating in the study.

### DNA Extraction

Areas of the tumor in which tumor cells represented >70% of all cells were extracted. Genomic DNA was extracted from paraffin-embedded tissues by use of xylene and alcohol. After digestion by proteinase K, DNA was extracted and purified by the phenol/chloroform/isoamyl alcohol method and precipitated in ethanol, as previously described[20].

### Bisulfite modification

Bisulfite modification of DNA results in conversion of unmethylated cytosine residues into uracil, whereas, the methylated cytosine residues, remains unchanged.

Briefly, 2 μg of DNA was denatured in 3 mol/L NaOH (2 μL) for 10 min at 50°C and then modified by adding a 500 μl of a freshly prepared bisulfite solution (2.5 M sodium bisulfate and 125 mM hydroquinone) following to an incubation for 12 h at 50°C. DNA samples were desalted through a column (Wizard DNA Clean-Up System, Promega), and then treated with 3 mol/L NaOH (5 μL) for 10 min at 37°C, followed by precipitation with 75 μl ammonium acetate (5 M). The pellet was washed with 2.5 volumes of ethanol, dried, and re-suspended in 20 μl tris (pH 8.0, 5 mM), then stored at -70° until used for MSP.

### Methylation-Specific PCR (MSP)

After bisulfite treatment, DNA samples were assayed by methylation-specific PCR. The PCR mixture was prepared in a 25 μl volume containing 1× buffer (Finzymes, Finland) with 2 mmol/L of MgCl_2_, 500 nmol/L of each primer (previously described sequences[20]), 0.2 mmol/L of dNTPs, 1 U of Hot Start Taq polymerase (Finzymes, Finland), and bisulfite-modified DNA. DNA amplification was performed in a thermocycler (Perkin-Elmer Corp.). The PCR amplification consisted of an initial hot-start step at 95°C for 10 min, followed by 40 cycles (45 s at 95°C, 45 s at 60°C, 60 s at 72°C), and a final 10 min extension at 72°C. Each MSP was repeated at least twice. In addition to a negative control, DNA from L132 and H1299 cells were used as a positive control for unmethylated and methylated alleles, respectively. PCR products were loaded on 2.5% agarose gel and 6% poly-acrylamide gel, stained with silver nitrate dyes and visualized under UV illumination.

### Immunohistochemistry

Paraffin embedded sections (3 μm thick) of esophageal tumor and adjacent normal tissue were used to perform IHC reaction. Briefly, the sections were mounted on poly-L-lysine-coated slides and dried at 60°C for 1 hour. The sections were dewaxed and rehydrated in a xylene-ethanol series and boiled in the Target Retrieval Solution of Dako (Dako, Carpinteria, CA, USA) in a microwave oven for 40 min. After endogenous peroxidase blocking, the following antibodies (Abs) were used: primary mouse monoclonal *p16*^*INK4a *^antibody (C-20) (Santa Cruz Biotechnology, Santa Cruz, CA, USA) (IgG2a, 200 μg/ml) at a working dilution of 1/1600, at 4°C overnight; mouse anti-human *p53 *monoclonal antibody (clone: DO-7, isotype IgG2b) (Dako, Carpinteria, CA, USA), incubation for 45 min at 37°C with a 1:50 dilution; and, for *MDM2*, IgM mouse monoclonal clone 1B10 (carboxy terminus of *MDM2*) was used (Novocastra Laboratories, New Castle, UK) with a 1:100 dilution, incubated overnight. After two washes in PBS, sections were incubated with EnVisionTM+System/HRP, Rabbit/Mouse (DAB+) (DakoCytomation, Carpinteria, CA, USA), a secondary antibody. The immunoreactivity was detected using diaminobenzidine (DAB) (DakoCytomation, Carpinteria, CA, USA) as the final chromogen. Finally, sections were counterstained with Meyer's Hematoxylin, dehydrated through a sequence of increasing concentrations of alcoholic solutions and cleared in xylene. During each IHC assay, proof slides were coupled with negative control slides on which the primary antibody was omitted. *P16*-positive and *p53*-positive esophageal squamous cell carcinoma was used as positive controls in every section. The cutoff values for abnormal expression were determined as follows: *MDM2 *> 30% [25]; *p53 *> 5% [26]; *p16 *< 5% [9].

### Statistical analysis

The Statistical Package for the Social Sciences software version 16.0 (SPSS Inc., Chicago, IL, USA) was used for statistical analyses. The correlation between two variables was evaluated using Pearson's chi-square and Fisher's exact tests. Survival rates were calculated by the Kaplan-Meier method. Using the log-rank test, we compared the survival of ESCC patients according to various clinicopathological factors. A 2-sided P value < 0.05 was considered as the significant statistical level.

## Results

Fifty ESCC patients (ages ranging from 35 to 83 years; with the mean age of 59.02 ± 11.41 years) were enrolled in this study. Twenty five (50%) patients were men and 25 were women with the male to female ratio of 1. Tumor sizes ranged from 2 to 12 cm, with a mean diameter was 4.97 ± 2.13 cm. Clinicopathological characteristics of the ESCC patients are summarized in table 1.

**Table 1:** Distribution of *p16 *methylation status according to clinicopathological features and protein expressions in ESCC tumors

	Number (%)	methylated	unmethylated	p value
**Age**				
<60	25 (54.3)	16 (59.3)	9 (47.4)	0.421
≥60	21 (45.7)	11 (40.7)	10 (52.6)	
**Gender**				
Male	25 (50)	15 (48.4)	10 (52.6)	0.773
Female	25 (50)	16 (51.6)	9 (47.4)	
**Histology**				
Well	25 (55.6)	15 (33.3)	10 (22.2)	0.211
Moderate	13 (28.9)	9 (20.0)	4 (8.8)	
Poor	7 (15.6)	2 (4.4)	5 (11.1)	
**Tumor site**				
Upper	1 (2.8)	0 (0.0)	1 (5.9)	0.854
Middle	23 (63.9)	12 (63.2)	11 (64.7)	
Lower	12 (33.3)	7 (36.8)	5 (29.4)	
**Tobacco use**				
Positive	14 (30.4)	8 (29.6)	6 (31.6)	0.896
Negative	32 (69.6)	19 (70.4)	13 (68.4)	
***p16 *protein**				
Positive	22 (44)	10 (32.3)	12 (63.2)	**0.033**
Negative	28 (56)	21 (67.7)	7 (36.8)	
**p53 protein**				
Positive	31 (62)	23 (74.2)	8 (42.1)	**0.020**
Negative	19 (38)	8 (25.8)	11 (57.9)	
**MDM2 protein**				
Positive	21 (42)	16 (51.6)	5 (26.3)	0.080
Negative	29 (58)	15 (48.4)	14 (73.7)	

**Figure 1:**
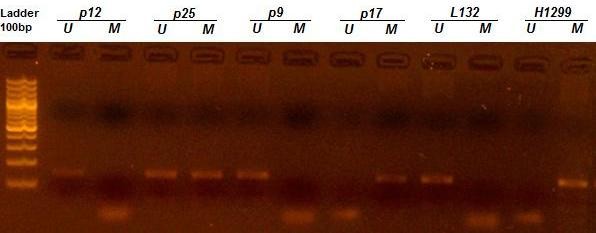
**MSP analysis of p16 gene in ESCC patients**. DNA from H1299 cells served as a positive control for hypermethylated DNA and L132 as a positive control for unmethylated DNA. Patient 17 (p17) and patient 25 (p25) were hypermethylated, which revealed 150 bp bands (M) with hypermethylated primers. Patient 12 (p12) and patient 9 (p9) were not methylated, having only unmethylated band (U).

The methylation status of 5' CpG island of the *p16 *gene was detected in 31/50 (62%) esophageal tumor samples, while two of the adjacent normal mucosa were methylated (P < 0.001). No significant association was found between the methylation status of the *p16 *gene and the factors studied, including age, sex, tumor histopathology, tumor site and size, and opium and tobacco use (cigarette and hookah smoking, Nass chewing) (Table 1). Figure 1 represents MSP analysis of *p16 *gene.

### Immunohistochemical staining

**Figure 2:**
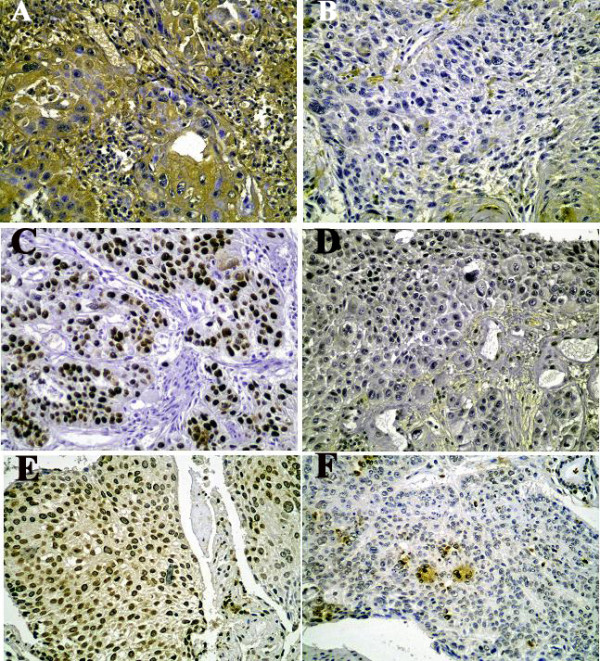
**Typical images of Immunohistochemical staining**. **a) **Positive *p16 *immunoreactivity. **b) **Negative *p16 *immunoreactivity **c) **Overexpression of *p53 *protein. **d) **Negative *p53 *immunostaining **e) **Overexpression of *MDM2 *protein. **f) **Negative *MDM2 *immunoreactivity.

IHC staining of *p16*, *p53 *and *MDM2 *are represented in figure 2.

#### a) Immunohistochemical staining of *p16*, *p53 *and *MDM2*

The negative expression of the *p16 *protein was detected in 28/50 (56%) tumors and only 7/50 (14%) normal tissues (P < 0.001).

The positive expression of *p53 *and *MDM2 *proteins in the nuclei was detected in 31/50 (62%) and 21/50 (42%) tumor tissues and in 7/50 (14%) and 30/50 (60%) normal esophageal tissues, respectively. There was a significant difference between tumor and normal tissues for *p53 *staining (P = 0.001), but not for *MDM2*. The histological grade of differentiation was not associated with the IHC staining of *p16*, *p53 *and *MDM2*.

#### b) Concomitant *p16*, *p53 *and *MDM2 *protein expression in ESCC

The overexpression of *MDM2 *was not significantly associated with the abnormal expression of *p53*. There was neither significant correlation between *p16 *and *p53*, nor between *p16 *and *MDM2 *immunoreactivity in esophageal tumor and normal samples, independently. Concomitant abnormal accumulation of the *p53 *protein with either *MDM2 *overexpression or abnormal underexpression of *p16 *was significantly more frequent in tumoral tissue compared to normal tissue; *p16-/p53+ *immunostaining was detected in 20/50 (40%) tumors and in none of the normal tissues; whereas the opposite combination (*p16+/p53-*) was found in 11/50 (22%) tumors and 36/50 (72%) normal specimens, with a statistically significant difference between tumor and normal samples in these subgroups (P < 0.001). In tumoral and normal tissues, the proportion of *p53+/MDM2*+ was 16/50 (32%) and 2/50 (4%), while *p53-/MDM2- *was detected in 14/50 (28%) and 14/50 (28%) respectively, with a statistically significant difference (P < 0.001). On the other hand, with *MDM2+/p16-*, there was not a significant difference in tumors compared to normal tissues (P = 0.078).

We also investigated the association between the *p16/p53/MDM2 *profile and tobacco use, histology of tumor, and *p16 *methylation status in ESCC. The samples were divided into four groups according to the number of alterations detected. The *p16-/p53+/MDM2+ *immunoprofile was only observed in the tumor tissues and not in the normal adjacent tissue (P < 0.001). Similarly we observed this profile more frequently in *p16*-methylated tumors (P = 0.035). We did not find any significant association between tobacco consumption and different histological subtypes with the different immunoprofiles. (Table 2)

**Table 2:** Correlation of *p16*/*p53*/*MDM2 *immunoprofile in ESCC patients with *p16 *methylation and clinicopathological features

	Alteration				
	
	a	b	c	d	p value
***p16 *methylation**					
Positive	3 (33.3)	6 (46.2)	12 (70.6)	10 (90.9)	**0.028**
Negative	6 (66.7)	7 (53.8)	5 (29.4)	1 (9.1)	
**Tobacco use**					
Positive	2 (22.2)	7 (58.3)	3 (21.4)	2 (18.2)	0.145
Negative	7 (77.8)	5 (41.7)	11 (78.6)	9 (81.8)	
**Histology**					
Well	3 (33.3)	7(53.8)	6 (50)	9 (81.8)	0.173
Moderate	2 (22.2)	4 (30.8)	5 (41.7)	2 (18.2)	
Poor	4 (44.4)	2 (15.4)	1 (8.3)	0 (0.0)	
**Tissue**					
Tumor	9 (56.2)	13 (43.4)	17 (80.9)	11 (100)	**0.001**
Normal	7 (43.8)	17 (56.6)	4 (19.1)	0 (0.0)	

^a ^*p16*^+^/*p53*^-^/*MDM2*^- ^profile

^b^*p16*^-^/*p53*^-^/*MDM2*^- ^profile or *p16*^+^/*p53*^+^/*MDM2*^- ^profile or *p16*^+^/*p53*^-^/*MDM2*^+ ^profile

^c^*p16*^-^/*p53*^+^/*MDM2*^- ^profile or *p16*^-^/*p53*^-^/*MDM2*^+ ^profile or *p16*^+^/*p53*^+^/*MDM2*^+ ^profile

^d^*p16*^-^/*p53*^+^/*MDM2*^+ ^profile

### Correlation between *p16*, *p53 *and *MDM2 *immunoreactivity, and hypermethylation of *p16*

Twenty one out of thirty one (67.7%) methylation-positive esophageal tumors showed a complete lack of immunoreactivity of *p16*. Twelve out of nineteen (63.2%) of unmethylated tumors represented diffuse immunoreactivity, whereas 7/19 (36.8%) of unmethylated tumors were not immunostained for *p16*. *p16 *negative staining was significantly correlated with *p16 *methylation (P = 0.033). Significant association was observed between abnormal accumulation of *p53 *and *p16 *hypermethylation (p = 0.020). Moreover, *p16 *methylation was more frequent in cases with concomitant accumulation of *p53 *and loss of *p16 *proteins i.e. 17/20 (85%), compared to the cases with *p16 *expressed and *p53 *negative, 4/11 (36.36%) (P = 0.01). *p16 *methylation was not associated with *MDM2 *overexpression. (Table 1)

### Patients' Survival

The 6-months, 1- year, and 2-year survival rates were 78%, 43%, and 28% in tumors with *p16 *hypermethylation and 43%, 14%, and 0% in tumors without *p16 *hypermethylation, respectively. The median survival duration of patients with *p16 *hypermethylation was 8 months, whereas it was 5 months for patients without hypermethylation. The 6 months, 1-year, and 2-year survival rates were 67%, 33%, and 33% in cases with *p16 *expression group and 62%, 31% and 18% in the cases with loss of *p16*, respectively. The median survival duration of patients with and without loss of *p16 *expression was 8 and 10 months, respectively.

There was no statistically significant difference in survival rates based on the *p16 *hypermethylation status or *p16 *protein expression.

## Discussion

Golestan province in northeastern Iran has a high incidence of ESCC, which is well above the world average. To better understand some aspects of the complex genetic/epigenetic alterations in this high incidence region, we investigated the role of some components of *p53*-*MDM2 *and *p16*-*Rb *pathways of cell cycle regulation and their possible cross-talk in ESCC tumorigenesis. Thus, we assessed *p16 *hypermethylation as an important mechanism of gene silencing, its impact on the *p16 *protein expression, along with its correlation with the *p53 *and *MDM2 *protein expression.

We showed that *p16 *protein expression was significantly associated with methylation of the *p16 *gene, indicating that *p16 *methylation may play a critical role in the silencing of this important tumor suppressor gene. On the other hand, significant differences in both methylation and loss of protein expression of *p16 *in tumoral tissue compared to the normal tissue confirms the critical role of these genetic and epigenetic alterations in the development of ESCC in this high-incidence region. In this study, *p16 *hypermethylation was observed in 62% of the patients. Previous reports of *p16 *methylation varied between 40-90% among ESCC patients in the Far East. Xing et al [27] studied 40 ESCC patients and detected the *p16 *gene hypermethylation in 40% of tumor samples. Whereas, Hardie et al [28] and Hibi et al [29] reported promoter methylation of the *p16 *gene in 85% (18/21) and 82% (31/38) of esophageal carcinoma, respectively. Wang et al [30] showed the aberrant hypermethylation of *p16*, as a frequent event, in 88% of ESCC patients in China. Abbaszadegan et al reported 73.3% *p16 *gene methylation in ESCC samples in Khorasan, another province in the northeastern Iran. In support of their data, this study indicates the critical role of *p16 *methylation in ESCC development in this high risk region [19].

In this study, we also examined *p16 *methylation in normal tissue adjacent to tumor, in order to clarify whether *p16 *methylation may have been occurred in the background of tumors. We showed that in two ESCC patients, *p16 *methylation occurred not only in the tumoral cells but also in the corresponding normal tissue. In the normal tissue of one of the two, *p16 *protein was not expressed. In line with our data on *p16 *methylation in normal tissue, Hibi et al presented one case of *p16 *methylation in the normal tissue in addition to WBCs of peripheral blood. They suggested that individuals with *p16 *promoter methylation of normal tissue might be prone to development of cancer. Furthermore, other studies have provided evidence of *p16 *methylation in normal tissue. *p16 *methylation of normal tissues, as a rare event, was also shown by Esteller et al [31]. Guan et al [32]reported the *p16 *methylation of transitional mucosa in 2/8 colon cancer patients. We can speculate that the presence of *p16 *methylation in normal tissue of these two studied ESCC patients may prone them to trigger tumor formation in these tissues. Although *p16 *hypermethylation most often occurs in the late preneoplastic stages (i.e. dysplasia) [33], it seems that in a small proportion of individuals, methylation may rarely occur in the normal tissue of esophagus. Environmental factors, previously reported as influencing aberrant hypermethylation, such as exposure to carcinogens or dietary factors, [34-37], along with a possible genetic predisposition, such as DNA methyltransferase activation [38], may be responsible for the epigenetic alterations and methylation induction in the normal tissue of this small proportion. However, further large-scale studies are required to focus on this issue and validate the probable impact of life-style and environmental factors and possible genetic predispositions on the methylation status of normal esophageal tissue.

Regarding *p16 *immunostaining, we did not detect hypermethylation in 7 out of 28 ESCC tumor tissues (25%) with negative *p16 *immunostaining. This suggested that other molecular mechanisms, such as point mutation, homozygous deletion or loss of heterozygosity, may contribute to *p16 *gene inactivation [39-41]. In 20% of ESCCs (10/50), *p16 *protein expression was accompanied by positive *p16 *hypermethylation. This can possibly be explained by hemi-methylation of the *p16 *gene. It may also reflect the high sensitivity of the MSP method, i.e. 0.1% methylated DNA, which would indicate that tumor samples with a low proportion of methylated DNA could be considered as methylation positive by MSP even though they may withhold *p16 *immunoreactivity due to unmethylated tumor cells in the same sample [42].

It has been hypothesized that *p53 *alterations may concomitantly occur with alterations in *p16*^*INK4a *^in carcinogenesis [43]. As for the *p53*-*MDM2 *pathway, when the *p16 *methylation status was compared with the *p53 *and *MDM2 *protein expression in ESCC patients, we observed that ESCC tumors with *p16 *epigenetic inactivation more often harbored increased levels of *p53 *protein expression. To our knowledge, this is the first report indicating the association between *p16 *hypermethylation and *p53 *protein accumulation in ESCC. Lee et al detected abnormal accumulation of *p53*, along with *p16 *promoter hypermethylation in colon cancer despite the inverse correlation between them as reported by other previous studies [44]. Esteller et al showed that *p53 *overexpression was independent of *p16 *methylation status in colorectal cancer [45]. Ishii et al reported more extensive DNA methylation in neoplastic lesions of ESCC with a *p53 *mutation than in those with wild-type *p53*, when assessing the promoter hypermethylation of 14 tumor suppressor genes; however *p16 *hypermethylation was not independently associated with *p53 *mutation[46].

These observations may help us address the occurrence of combined molecular mechanisms, which are likely to play a major role in ESCC tumor progression. Since cell populations of the primary neoplasm are heterogeneous, a single marker cannot specifically and accurately predict the behavior of the tumor [47]. Abnormal *p53 *expression occurs concomitantly with abnormally expressed *p16 *or *MDM2 *proteins in the tumor. On the other hand, *p16 *hypermethylation is associated with a larger accumulation of abnormal protein expression in both *p16*-*Rb *and *p53*-*MDM2 *pathways. All these findings in addition to correlation between *p16 *hypermethylation and *p53 *abnormal accumulation, indicate a possible overlap and cross-talk between the involved pathways.

Although it has been reported that *p16 *hypermethylation is a predisposing epigenetic trait in the familial ESCC in Iran [21], the role of other factors such as environmental factors has not yet been ruled out. It has been reported that high-level exposure to polycyclic aromatic hydrocarbons may contribute to the high incidence of ESCC in the northeastern Iran [4]. Concomitant *p16 *hypermethylation and *p53 *overexpression may be a consequence of various environmental, dietary or lifestyle factors peculiar to this region, associated with an increased susceptibility to ESCC. However due to the lack of a precise evaluation of environmental exposures in this study, we could not strongly deduce any correlation between these factors and *p16 *methylation or protein expression status, as well as *p53 *and *MDM2 *overexpression.

## Conclusion

In summary, we conclude that *p16 *gene silencing caused by hypermethylation of CpG islands may be a major mechanism in the ESCC development. It is associated with p53 protein overexpression in the ESCC patients of northeastern Iran. This is the first study indicating the association between *p16 *hypermethylation and *p53 *protein overexpression. The impact of *p16 *hypermethylation on accumulation of abnormally expressed proteins in the *p53*-*MDM2 *pathway, along with the observed concomitant accumulation of *p53 *with either *MDM2 *overexpression or decreased *p16 *expression, suggests a possible cross-talk of the involved pathways in ESCC development in northeastern Iran. Further studies of the methylation status of various cancer-related genes in a large sample size, accompanied by the assessment of exposure to the potentially harmful environmental factors, are needed to better elucidate the genetic changes occurring in ESCC carcinogenesis and tumor progression in this high risk population.

## Competing interests

The authors declare that they have no competing interests.

## Authors' contributions

NT carried out data collection, the molecular genetic studies, designed the questionnaire, participated in designing the study, and drafted the manuscript. FB participated in the study design and coordination. MS participated in the study design and coordination, participated in data interpretation and carried out immunohistochemistry study. HK performed the statistical analysis, interpretation of data, and participated in drafting the manuscript. RM general supervision of the research group, management of data collection and questionnaire development, participated in the study design and coordination. OM participated in epigenetic study, interpretation of data, and scientifically revised the manuscript. BM participated in the immunohistochemistry study. AA carried out epigenetic studies. MRA conceived of the study, participated in the study design and coordination, and scientifically revised the manuscript. All authors read and approved the final manuscript.

## Pre-publication history

The pre-publication history for this paper can be accessed here:

http://www.biomedcentral.com/1471-2407/10/138/prepub
